# *Thalassiosira* spp. community composition shifts in response to chemical and physical forcing in the northeast Pacific Ocean

**DOI:** 10.3389/fmicb.2013.00273

**Published:** 2013-09-23

**Authors:** P. Dreux Chappell, LeAnn P. Whitney, Traci L. Haddock, Susanne Menden-Deuer, Eric G. Roy, Mark L. Wells, Bethany D. Jenkins

**Affiliations:** ^1^Department of Cell and Molecular Biology, University of Rhode IslandKingston, RI, USA; ^2^Graduate School of Oceanography, University of Rhode IslandNarragansett, RI, USA; ^3^School of Marine Sciences, University of MaineOrono, ME, USA

**Keywords:** *Thalassiosira*, iron, temperature, Haida eddy, community composition, automated ribosomal intergenic spacer analysis

## Abstract

Diatoms are genetically diverse unicellular photosynthetic eukaryotes that are key primary producers in the ocean. Many of the over 100 extant diatom species in the cosmopolitan genus *Thalassiosira* are difficult to distinguish in mixed populations using light microscopy. Here, we examine shifts in *Thalassiosira* spp. composition along a coastal to open ocean transect that encountered a 3-month-old Haida eddy in the northeast Pacific Ocean. To quantify shifts in *Thalassiosira* species composition, we developed a targeted automated ribosomal intergenic spacer analysis (ARISA) method to identify *Thalassiosira* spp. in environmental samples. As many specific fragment lengths are indicative of individual *Thalassiosira* spp., the ARISA method is a useful screening tool to identify changes in the relative abundance and distribution of specific species. The method also enabled us to assess changes in *Thalassiosira* community composition in response to chemical and physical forcing. *Thalassiosira* spp. community composition in the core of a 3-month-old Haida eddy remained largely (>80%) similar over a 2-week period, despite moving 24 km southwestward. Shifts in *Thalassiosira* species correlated with changes in dissolved iron (Fe) and temperature throughout the sampling period. Simultaneously tracking community composition and relative abundance of *Thalassiosira* species within the physical and chemical context they occurred allowed us to identify quantitative linkages between environmental conditions and community response.

## INTRODUCTION

Diatoms are unicellular, photosynthetic eukaryotes found throughout marine and freshwater environments (Round et al., 1990). They are important primary producers, believed to generate roughly 40% of the 45–50 billion tons of organic carbon fixed annually in the sea and up to 90% of the photosynthetically derived organic carbon fueling coastal ecosystems (Nelson et al., 1995). Diatoms are the most diverse group of phytoplankton, with an estimated 200,000 different species (Mann and Droop, 1996). Within the genus *Thalassiosira* alone, there are an estimated 100 different freshwater and marine species from a wide range of habitats (Round et al., 1990). *Thalassiosira* species are important contributors to marine primary production in temperate to polar regions (Karentz and Smayda, 1984; Degerlund and Eilertsen, 2010), where they can be a significant component of phytoplankton blooms (Haigh et al., 1992; Hoppenrath et al., 2007; Yoshie et al., 2010).

It can be challenging to elucidate the biogeography and the composition of complex diatom communities. Some diatoms have unique morphological features that enable them to be identified and enumerated with light microscopy and cell counts. Many other diatoms, including smaller *Thalassiosira* spp., cannot be visually distinguished by light microscopy (Tomas, 1997; Kaczmarska et al., 2009). Thus, it is difficult to assess the ecological importance of individual species when it is only possible to classify and enumerate them based on genus level data. Diatom species from the same genus can have distinct physiological properties that may have significant consequences for the environment. For example, *Thalassiosira oceanica* can grow at close to maximal growth rates in much lower iron (Fe) concentrations than do other members of the *Thalassiosira* genus (Sunda and Huntsman, 1995). There are also differences in the amount of nitrogen that various *Thalassiosira* species store when nitrogen is in excess (Dortch et al., 1984). These physiological differences within the Thalassiosiroids have ecological consequences for bloom formation in different oceanic regimes and for how different species respond to climatically modulated changes.

Given their importance to marine food webs, nutrient cycling, and global climate, there have been significant efforts to classify *Thalassiosira* species using genetic techniques (Medlin et al., 1996; Kaczmarska et al., 2005; Alverson et al., 2007). Genetic studies highlight discrepancies between morphological- and sequence-based classifications. For example, the Provasoli-Guillard National Center for Marine Algae and Microbiota strain CCMP1010 was originally identified as *T. pseudonana* on the basis of morphology and was reclassified as *T. weissflogii* when its rRNA sequence was obtained (Von Dassow et al., 2008). While the reclassification of CCMP1010 was also justified by a re-evaluation of its morphology, another *Thalassiosira* species, *T. oceanica *CCMP1004 remains morphologically classified as *T. oceanica*, while rRNA phylogeny supports that it is a strain of *T. rotula *(Von Dassow et al., 2008). *Thalassiosira* species are also becoming important model diatoms because of the increasing availability of genomic information, which currently includes the completed genomes of *T. pseudonana* (Armbrust et al., 2004) and *T. oceanica* (Lommer et al., 2012). An analytical tool that can rapidly distinguish among *Thalassiosira* species enables investigations of species distributions *in situ* and allows for a broader assessment of community responses to environmental and ecological change.

Here we examine *Thalassiosira* community composition in natural populations in the northeast Pacific Ocean using an assay we developed that distinguishes species and assesses diversity within the genus *Thalassiosira*. The assay uses polymerase chain reaction (PCR) amplification with primers specific for the Thalassiosiroid internal transcribed spacer region two (ITS2), the region between the 5.8S rDNA and the 28S rDNA. The length of the ITS2 region varies between species and the patterns of diagnostic lengths amplified from a given sample can be resolved in a capillary sequencer. This method, known as automated ribosomal intergenic spacer analysis (ARISA), was developed to rapidly compare microbial bacterial diversity (Fisher and Triplett, 1999) and has been applied to assess diversity in a number of different habitats (e.g., Dorigo et al., 2005; Danovaro et al., 2006). In the majority of ARISA studies, primers are designed to be inclusive of a broad range of taxonomic groups and the focus is not necessarily on identifying species, but on assessing the overall diversity of a population. While the application of ARISA to eukaryotic organisms is much more limited, it has been used to explore diatom diversity and community composition in two recent studies (Hubbard et al., 2008; Fechner et al., 2010). Similar to the Hubbard et al. (2008) ARISA method targeting the diatom genus *Pseudo-nitzschia*, we developed a *Thalassiosira*-specific ARISA method to both identify individual *Thalassiosira* species as well as to compare *Thalassiosira* community composition in different samples.

**FIGURE 1:**
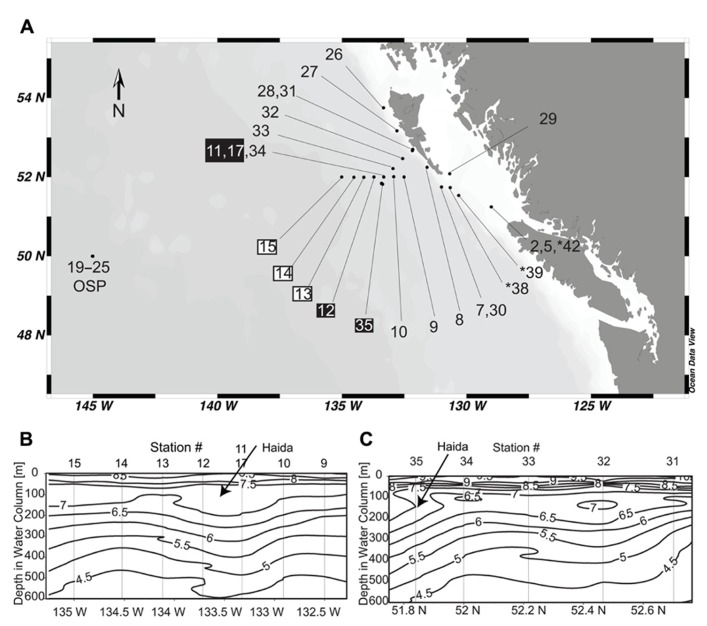
**(A)** Station locations for cruise T0206 on the R/V Thomas G. Thompson from May 12 to June 8, 2007. Stations from the eddy core are labeled with a black box and stations from the leading edge are labeled with a white box. Stations where ITS2 was sequenced, but ARISA analysis was not done because samples were collected with a different size cut-off filter are indicated by “*” symbol. **(B)** Temperature contours through the Haida eddy when first encountered (May 19–20, 2007). Station numbers corresponding to the labels in **(A)** are shown at the top of the contour plot. The arrow marks the shallow warm waters characteristic of Haida eddies. **(C)** Temperature contours through the Haida eddy on the return trip approximately 2 weeks later (June 3–4, 2007). The arrow marks the shallow warm waters characteristic of Haida eddies.

We used ARISA to assess *Thalassiosira* diversity in field samples collected from both nearshore and offshore regions of the northeastern Pacific Ocean (**Figure 1A**). Stations sampled included one of the longest running open ocean time-series stations, ocean station papa (OSP; Harrison, 2002) and two transects through a 3-month-old Haida eddy (Xiu et al., 2011). As it is well established that Fe inputs significantly impact phytoplankton biomass and species composition in these waters (Boyd et al., 2004; Johnson et al., 2005) and, more recently, that anticyclonic mesoscale Haida eddies may be a major mechanism for transporting Fe from shelf to offshore waters (Johnson et al., 2005; Xiu et al., 2011), measurements of dissolved Fe were included in our analyses. Correlations between ARISA data and environmental variables indicated that dissolved Fe and temperature were important drivers of shifts in Thalassiosiroid community composition among sampling locations. Our findings demonstrate the usefulness of ARISA-like methods for quickly identifying specific species that may be important players in a given ecosystem and enabling the comparison of species assemblages and environmental variables between regions.

## MATERIALS AND METHODS

### SAMPLE COLLECTION

**FIGURE 2:**
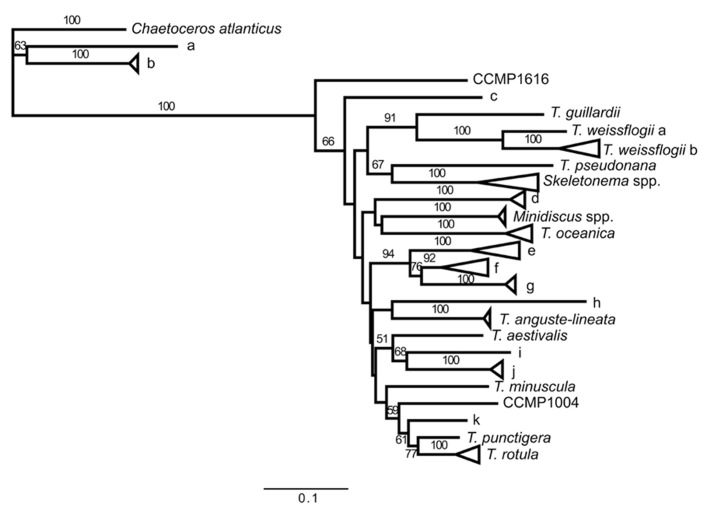
**Maximum likelihood tree of ITS2 sequences from GenBank^®^ and field samples generated in the sequence analysis program Geneious^®^ using the PHYML algorithm with the Jukes-Cantor substitution model and 1000 bootstraps.** Sequences with >90% identity at the sequence level have been condensed into groups. Branch labels indicate the type strain of sequences within a branch if there is a species that has previously been described in the group. Throughout the figure *Thalassiosira* is abbreviated as *T*. If the group does not include a type strain, the branch was given a letter (branches a–k). Information on the sequences in each branch is listed in**Table 2**. Bootstrap values greater than 50 are indicated.

Samples were collected on cruise T0206 on the R/V Thomas G. Thompson from May 12–June 8, 2007 (**Figure 1**). Approximately 1 L of surface water was collected at each station using the shipboard conductivity, temperature, depth (CTD) profiler rosette and filtered by pressure onto 25 mm diameter 0.2 μm polyethersulfone Supor^®^ 200 filters (Pall Corporation, USA) using a Masterflex^®^ peristaltic pump. In a few samples from OSP, water was collected via a towfish without corresponding CTD measurements. Water from the towfish was prefiltered through 10 μm polyester filters that were discarded resulting in only the 0.2–10 μm fraction of biomass being collected. Since these samples were processed differently, we included sequences from these samples in our phylogenetic analysis (annotated on the tree in **Table 2** and **Figure 2** as OSP). Samples filtered from the towfish were not included in ARISA analysis because of lacking corresponding environmental data and that a different size class of organisms was captured on the filters in comparison to the other stations. Likewise, samples from stations 38, 39, and 42 were filtered on 5 μm polyester filters, so the 0.2–5 μm fraction of biomass was not collected. Sequences from these samples were also included in the phylogenetic analysis but these samples were not considered in the ARISA analysis. Immediately following filtration, filters were transferred to screw-cap tubes containing 500 μl Qiagen^®^ AP1 buffer (Qiagen^®^, Germany), flash frozen in liquid nitrogen, and stored at -80°C until analysis.

### ENVIRONMENTAL DATA

Surface temperature, salinity, and fluorescence values were obtained from the Sea-Bird SBE-911+ CTD system attached to the sampling rosette. Surface (10 m) dissolved Fe (0.2-μm filtration) was determined as described in Roy et al. (2008). To briefly summarize the Roy et al. (2008) method, total Fe concentrations were determined by chemically reducing all dissolved Fe species to Fe(II) with sulfite. The amount of Fe(II) was determined using an automated flow injection-based FeLume system (Waterville Analytical) that measures the luminescence associated with the reaction between Fe(II) and an alkaline luminol solution.

### DNA EXTRACTION

Filters were extracted using the Qiagen^®^ DNeasy^®^ plant DNA extraction kit (Qiagen^®^, Germany) following the general protocol with one minor exception. To lyse the cells, a mixture of 0.1 and 0.5 μm silica beads was added to each tube with the RNase A enzyme, the tubes were then incubated at 65°C for 10 min, followed by bead beating for 1 min to enhance cell breakup. At the end of the protocol, samples were eluted in two elution steps into a total of 200 μl Qiagen® AE buffer and quantified using a NanoDrop^TM^ 8000 spectrophotometer.

### CLONING

The ITS2 region was amplified using degenerate PCR primers designed to amplify all of the ITS2 sequences of the Thalassiosiroids with an effort to avoid those from other closely related diatoms (forward primer: 5′-RCGAAYTGCAGAACCTCG-3′; reverse primer: 5′-TACTYAATCTGAGATYCA-3′). The PCR used the following conditions: 2–8 ng of template DNA, 500 nmol L^-1^ forward and reverse primers, 1× BIO-X-ACT^TM^ Short Mix (Bioline USA Inc., Taunton, MA, USA) and PCR-grade water were combined in a 25 μL reaction in PCR tubes. The PCR cycling conditions were: 95°C for 5 min; 35 cycles of: 95°C for 1 min, 46°C for 1 min, and 72°C for 30 s; followed by a final extension at 72°C for 10 min and cooling to 4°C. All PCRs were performed using a Mastercycler^®^ gradient PCR machine (Eppendorf AG, Hamburg, Germany).

Polymerase chain reaction products were observed by agarose gel electrophoresis and all positive PCRs were cleaned up using the QIAquick PCR purification kit (Qiagen^®^, Germany). In a few cases amplicons were gel extracted using the QIAquick gel extraction kit (Qiagen^®^, Germany) because multiple bands occurred well outside the range of expected ITS2 sizes, indicating amplification of a non-Thalassiosiroid ITS2 amplicon or significant quantities of primer dimers.

Purified amplicons (3 μL) were ligated into the pGEM^®^-T vector overnight at 4°C (Promega Corporation, Madison, WI, USA). Ligations were used to transform Z-Competent^TM^ *Escherichia coli* (Zymo Research Corporation, Irvine, CA, USA), plated onto Luria–Bertani (LB) agar plates with 100 μg mL^-1^ carbenicillin, 80 μg mL^-1^ 5-bromo-4-chloro-indolyl-galactopyranoside (X-gal), and 0.5 mmol L^-1^ isopropyl-β-D-1-thiogalactopyranoside (IPTG), and incubated overnight at 37°C. White colonies were picked onto another LB agar/carbenicillin/X-gal/IPTG plate and again incubated overnight at 37°C to confirm blue-white screening. Approximately five white colonies per PCR were grown overnight in liquid LB/carbenicillin at 37°C and plasmids were purified using the QIAprep spin miniprep kit (Qiagen^®^, Germany). Three stations (stations 5, 17, and OSP) were sequenced to greater depth (10–20 clones each). Purified plasmids were sequenced using the universal T7 primer (5′-TAATACGACTCACTATAGGG-3′) on an ABI 3130xl genetic analyzer (Life Technologies Corporation, Carlsbad, CA, USA) by the Rhode Island Genomics and Sequencing Center.

Sequences were trimmed using the Geneious Pro^TM^ software package (Biomatters, Auckland, New Zealand; Drummond et al., 2011), and aligned with all ITS2 sequences of *Thalassiosira*, *Minidiscus*, *Skeletonema*, and *Chaetoceros* species found in GenBank^®^ trimmed to the same region amplified by our degenerate ITS2 primers (excluding the primer region itself) using the MUSCLE algorithm (Edgar, 2004). The accession numbers for sequences obtained from other studies in GenBank^®^ which were included in the phylogenetic analysis are: AY660001, DQ280326, DQ469928, DQ897642, EF134953–4, EF208779–EF208794, EF208796, EF208799–EF208801, EF362633, ET018147, ET022313, ET029232, ET600777, FJ590769, FJ590771, and HQ685854. The MUSCLE alignment was examined by eye to fix any small errors and the PHYML algorithm (Guindon and Gascuel, 2003) with the Jukes–Cantor substitution model was used to generate a maximum likelihood tree with 1000 bootstraps.

### FRAGMENT ANALYSIS

Fragment analysis was done using a method similar to Hubbard et al. (2008) where PCR amplification used the forward degenerate ITS2 PCR primer (5′-RCGAAYTGCAGAACCTCG-3′) modified by the addition of a 5′ fluorescent FAM label (Life Technologies Corporation, Carlsbad, CA, USA) and the non-fluorescently labeled reverse degenerate ITS2 PCR primer listed earlier. For the fragment analysis PCR, 6 ng of DNA, 500 nmol L^-1^ primers, 1× BIO-X-ACT^TM^short mix (Bioline USA Inc., Taunton, MA, USA) and PCR-grade water were combined in a 25 μL reaction in PCR tubes. The PCR cycling conditions were: 95°C for 5 min; 32 cycles of: 95°C for 1 min, 46°C for 1 min, and 72°C for 30 s; followed by a final extension at 72°C for 10 min and cooling to 4°C. All PCRs were performed using a Mastercycler^®^ gradient PCR machine (Eppendorf AG, Hamburg, Germany).

FAM labeled PCR products were purified using ethanol precipitation in 1.5 mL tubes with 0.1 volumes of 3 mol L^-1^ sodium acetate and two volumes of 100% ethanol. The reaction was well mixed and incubated at -80°C for a minimum of 30 min. Tubes were then centrifuged (20,000 × *g*) for 45 min at 4°C in an Eppendorf 5810R centrifuge (Eppendorf AG, Hamburg, Germany). The supernatant was carefully decanted and the pellet was washed with 200 μL 70% ethanol and then with 200 μL 100% ethanol, being centrifuged (20,000 × *g*) for 5 min at 4°C after each wash. After decanting the final ethanol wash the tubes were dried completely at 37°C for 5–10 min and pellets were resuspended in 50 μL PCR-grade water. One microliter of resuspended sample was mixed with 0.3 μL GeneScan^TM^ 600 LIZ^®^ size standard (Life Technologies Corporation, Carlsbad, CA, USA) and 10.7 μL Hi-Di formamide and run on an ABI 3130xl genetic analyzer (Life Technologies Corporation, Carlsbad, CA) by the Rhode Island Genomics and Sequencing Center. Fragment profiles were analyzed using Peak Scanner^TM^ v1.0 software.

### CLUSTER ANALYSIS

Comparative analysis of fragments from all samples was done in a manner similar to Nelson (2009). Peak height and size were determined for each FAM peak between 320 and 420 bases on every fragment run and binned into the fragment lengths listed in **Table 1**. If peak heights were off-scale, the PCR product was re-run at a 1:2 or 1:10 dilution. If the majority of peaks were below 1000 in peak height, and there were no peaks dominating the profile that would end up offscale, the PCR product was re-run with twice to four times as much sample added to the fragment analysis mixture and re-analyzed. Data for each sample were normalized to total peak height and all analyses were done using relative peak heights (the height of each peak relative to the overall height of peaks in that sample). A relative abundance matrix was generated combining the relative peak height data for all samples at all fragment lengths where a peak was detected. Samples that did not have a measurable peak for one of the fragment lengths were recorded as zero. All subsequent analyses were done using PRIMER v.6 (Primer-E Ltd, Plymouth, UK; Clark, 1993). Fragment analysis data from multiple ARISA runs (duplicate PCR runs and/or duplicate fragment analysis runs) were averaged for each sample. The Shannon–Wiener index of diversity was computed on the relative abundance data for each station. For ARISA samples, a similarity matrix of Bray–Curtis coefficients was used to compare the relative abundance data and to establish a cluster dendrogram using the “group average” mode of clustering. Fragment analysis profiles of replicates were also used to generate a Bray–Curtis coefficient similarity index and produced the same results as the averages (data not shown). To determine which single environmental variable or group of environmental variables (temperature, salinity, dissolved Fe, fluorescence, and bottom depth) best explained the similarity distribution of these data, the environmental data and Bray–Curtis similarity matrices were analyzed using the BEST analysis with the BIOENV algorithm using the Spearman rank correlation method and D1 Euclidean distance as the resemblance measure. Additionally, we ran a non-parametric (Spearman) correlation on all of the environmental variables against each other and report the Spearman correlations (ρ) and the two-tailed *p* values. To evaluate whether autocorrelation of variables impacted our results, we reran the BEST analysis and individually removed correlated variables.

**Table 1:** Fragment sizes of 5.8S-ITS2 sequences in fragment analysis and their associated labels in **Figure 2**.

Size of fragment (bases)	Branch label in **Figure 2**	Group label in Figure **4**
348–349	*T. oceanica*	1
357–358	N/A	2
362–363	N/A	3
366–367	d	4
368–369	*Skeletonema*	5
374–375	*Minidiscus, Skeletonema*	6
377–378	CCMP1616, *Skeletonema*	7
384–385	e, g, *T. minuscula*	8
386–387	f, i, j, *T. puntigera, T. rotula, Skeletonema*	9
388–389	h, k, *T. anguste-lineata*, CCMP1004	10
390–391	*T. aestivalis*	11
393–394	*T. guillardi, T. pseudonana*	12
396–397	*T. weissflogii*	13
402–403	N/A	14
404–405	C	15
409–410	*C. atlanticus*, a, b	16

## RESULTS

### STUDY SITE

On a 1-month cruise in the northeastern Pacific Ocean (**Figure 1A**), we collected biological samples from coastal stations (2, 5, 7, 8, 26, 27, 28, 29, 30, 31, 38, and 39), two offshore transects (stations 9–15 and stations 31–35) that crossed through a juvenile (3-month-old) Haida eddy, and multiple samples at the high nitrate low chlorophyll (HNLC) time-series station, OSP. The details of the Haida eddy are described elsewhere (Xiu et al., 2011). The depth profiles of temperature for the first transect through the Haida eddy (May 18–19, 2007) showed a vertically structured water column (**Figure 1B**). Stations 11 and 12 represent the center of the eddy as indicated by the warming of waters below 100 m when compared to surrounding stations, which is characteristic of a Haida eddy (Crawford, 2002). Station 17 was a reoccupation of station 11 approximately 1 day later. The depth profiles of temperature of the second transect through the Haida eddy 2 weeks later (June 3–4, 2007) show that the center of the eddy moved slightly southwest of its previous location (station 34) and was subsequently located at station 35 (**Figure 1C**).

### PHYLOGENETIC ANALYSIS OF 5.8S-ITS2 SEQUENCES

The GenBank^®^ accession numbers for the sequences generated in this study are: JQ044517–JQ044679. A maximum likelihood tree of 5.8S-ITS2 sequences of samples from this cruise, isolates in the Jenkins laboratory diatom collection, and Thalassiosiroid sequences available in GenBank^®^ shows that sequences resembling *T. oceanica* were found at almost all stations (Branch “*T. oceanica*”: **Figure 2**; **Table 2**). *T. oceanica* was a dominant portion the *Thalassiosira* sequence library composition at stations along the two transects off Haida Gwaii (stations 9–17 and 31–35) as well as in the center of the Haida eddy (stations 11, 12, 17, and 35). In contrast, OSP samples were dominated by a single as yet uncultured species of *Thalassiosira* (Branch “g”: **Figure 2**; **Table 2**). Our sequence library data showed that the *Thalassiosira* ITS2 primers sometimes amplified sequences from other closely related centric diatoms including *Skeletonema* spp. (12%), *Minidiscus* spp. (4%), and *Chaetoceros* spp. (6%), but the majority of our sequence data (78%) was associated with *Thalassiosira* species (**Figure 2**; **Table 2**).

**Table 2:** List of sequences associated with each phylogenetic branch shown in **Figure 2**.

Branch label	Sequences
*C. atlanticus*	*Chaetoceros atlanticus*, Stn 25(1)
a	Stn 25(1)
b	Stn 25(2)
CCMP1616	CCMP1616 (*Thalassiosira oceanica* morphology)
c	Stn 38(1)
*T. guillardii*	*Thalassiosira guillardii *(CCMP988)
*T. weissflogii* a	*Thalassiosira weissflogii* (CCMP1051)
*T. weissflogii* b	*Thalassiosira weissflogii* (CCMP:1010,1047,1048,1050,1052,1053,1336,1587; BILB2001)
*T. pseudonana*	*Thalassiosira pseudonana* (CCMP:1011,1012,1014,1015,1335)
*Skeletonema* spp.	*Skeletonema* sp. GFC-2005, Stn 2(3), Stn 5(3), Stn 7(2), Stn 26(2), Stn 29(2), Stn 42(5)
d	Stn 11(1), Stn 39(2)
*Minidiscus* spp.	*Minidiscus tricolatus, Minidiscus* sp. CCL-2009, Stn 2(1), Stn 27(1), Stn 30(2), Stn 38(1), Stn 39(1)
*T. oceanica*	*Thalassiosira oceanica* (CCMP:999,1001,1005,1006), Stn 2(2), Stn 5(2), Stn 7(4), Stn 8(4), Stn 9(4), Stn 10(5), Stn 11(4), Stn 12(5), Stn 13(4), Stn 14(6), Stn 15(4), Stn 17(2), Stn 25(1), Stn 26(2), Stn 27(4), Stn 30(1), Stn 32(5), Stn 33(5), Stn 34(6), Stn 35(4)
e	Stn 17(1), Stn 19(1)
f	Lab isolate Th-6, Stn 5(1), Stn 19(1)
g	OSP (19), Stn 32(1), Stn 35(1)
h	Stn 38(1)
*T. anguste-lineata*	*Thalassiosira anguste-lineata*, Stn 5(3)
*T. aestivalis*	*Thalassiosira aestivalis*
i	Stn 30(1)
j	Lab isolate B-A1, Stn 26(1)
*T. minuscula*	*Thalassiosira minuscula* (CCMP1093)
CCMP1004	CCMP1004 (*Thalassiosira oceanica* morphology)
k	Stn 2(1)
*T. punctigera*	*Thalassiosira punctigera*
*T. rotula*	*Thalassiosira rotula* (CCMP:1018, 3096), Stn 26(1)

*Values in parentheses following station numbers represent the number of sequences of that type recovered in the sequences from that station.*

### FRAGMENT ANALYSIS OF AMPLIFIED ITS2 REGION

**FIGURE 3:**
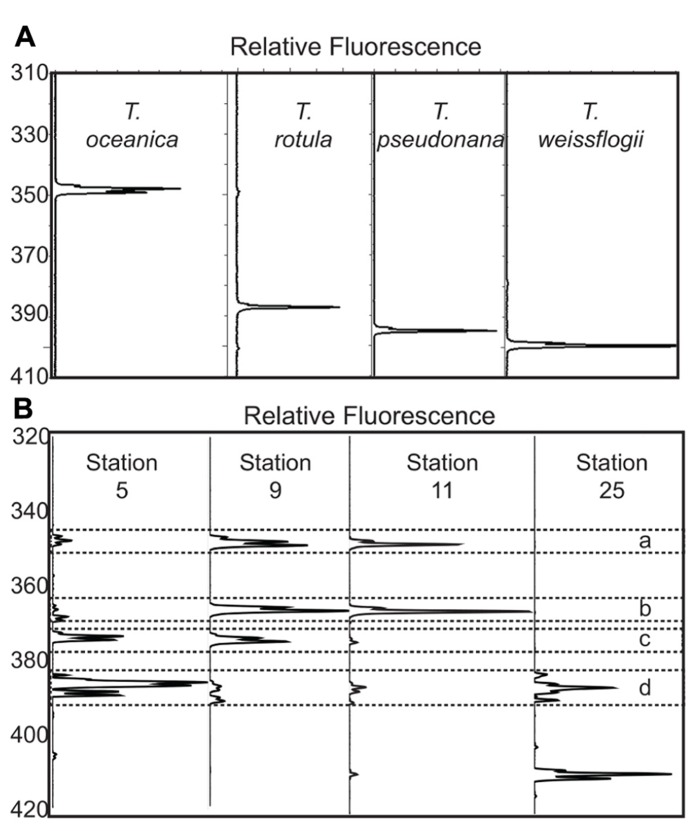
** (A)** Fragment analysis plots from four cultured isolates of *Thalassiosira* (*T. oceanica* CCMP1005, *T. rotula *CCMP3096, *T. pseudonana* CCMP1335, and *T. weissflogii* CCMP1010). The *x*-axis for each trace is the fluorescence scaled to the largest peak for each sample. The *y*-axis is the length in bases associated with each peak, as determined by the 600-LIZ^®^ standard. **(B)** Example of fragment analysis plots from four representative stations. The axes are the same as in 3A. Station 5 is a coastal station close to Vancouver Island, station 9 is a station at the coastal edge of the transect off Haida Gwaii, station 11 is at the center of the Haida eddy, and station 25 is from OSP. The dashed box marked “a” is surrounding the peaks at 348–349 bases associated with *T. oceanica*. The dashed boxes marked “b” surrounds peaks at 366–367 bases, which is associated with an unidentified *Thalassiosira* sp. for which we have ITS2 sequence data. The dashed box marked “c” surrounds the peaks at 374–375 bases associated with *Minidiscus* spp. The dashed box marked “d” surrounds the peaks between the sizes of 384 and 389 bases that are not associated with phylogenetically distinct species (**Table 2**).

In addition to validating ARISA fragment length identity by comparison to our 5.8S-ITS2 sequence database, the assignment of ARISA peaks was verified by amplifying the 5.8S-ITS2 region from cultured isolates from the National Center for Marine Algae and Microbiota (NCMA, formerly the CCMP): *Thalassiosira oceanica* CCMP1005, *Thalassiosira pseudonana* CCMP1335, *Thalassiosira weissflogii* CCMP1010, and *Thalassiosira rotula* CCMP3096, isolated by T. Rynearson on this cruise. The resulting ARISA electropherograms revealed distinct peaks corresponding to expected ITS2 sequence lengths (**Figure 3A**). The length of the amplicon region in sequences from NCBI and field samples varied by 60 bases, and there were 16 distinct length groupings among the different species (**Table 1**). In some cases, the amplicon length is distinct at the species level (e.g., groups 1, 4, 11, 13, 15, and 16). Some fragment lengths are associated with two species; e.g., group 6 is associated with both *Minidiscus* spp. and a *Skeletonema* sp. In our field samples, the majority of 375 base length sequences can likely be assigned to *Minidiscus* spp., since only one of the seven 375 base sequences returned was a *Skeletonema* sp. sequence. Most of the *Skeletonema* spp. sequences in our database had a fragment length of 369 bases. There were also ITS2 amplicon lengths associated with multiple species of *Thalassiosira* (e.g., groups 8–10).

**FIGURE 4:**
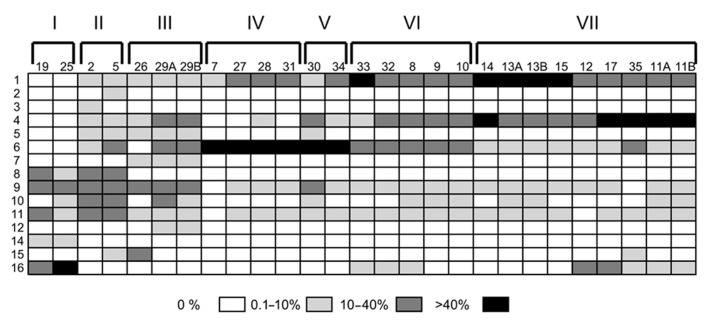
**Relative abundance of fragments from all stations.** Each column represents a different sample with its station identifier listed at the top of the column. Each row represents a different fragment group as listed in **Table 1**. Group 13 was not found in any samples and is thus not included in the figure. The shading of the different boxes indicates the percentage of the total peak height that each fragment group was responsible for in a given sample. Samples are ordered based on Bray–Curtis clusters with roman numerals corresponding to the clusters as identified in **Figure 5**.

Electropherograms from field samples with mixed populations of *Thalassiosira* species also showed well-resolved peaks centered on different fragment lengths, though multiple peaks were present per sample (**Figure 3B**). Comparing the relative abundance of the different fragment lengths at all stations demonstrates clear community composition shifts between different sampling locations (**Figure 4**). The shallow coastal stations (stations 2, 5, 26, and 29) had the most peaks, the open ocean stations (stations 9–25) had fewer peaks, and the stations at the shelf break along Haida Gwaii (stations 7, 27, 28, and 31) had the fewest peaks (**Figure 4**). It is also evident that at most of the stations, except the shallow coastal stations (stations 2, 5, 26, and 29), one or two fragment lengths dominated most of the fragment profiles with the other peaks being very minimal (**Figure 4**).

The Shannon–Wiener diversity index (*H*′) and evenness (*J*′) of the relative abundance ARISA data were positively correlated (**Table 3**). The highest diversity was seen in the shallow coastal stations (stations 2, 5, 26, and 29). The lowest diversity was seen in the samples from the deeper but still relatively coastal region off of Haida Gwaii (stations 7, 27, 28, and 31).

**Table 3:** Statistical measurements of relative abundance data.

Station	*H*′(log_**10**_)	S	*J*′
5	0.77	10	0.77
29B	0.75	9	0.78
29A	0.74	9	0.77
32	0.71	6	0.91
26	0.70	9	0.73
2	0.67	9	0.70
8	0.66	7	0.78
30	0.64	7	0.76
17	0.62	6	0.80
10	0.62	6	0.80
33	0.62	6	0.79
12	0.62	5	0.88
9	0.61	6	0.79
19	0.59	5	0.85
35	0.58	6	0.74
25	0.57	6	0.73
11A	0.56	7	0.66
13B	0.56	7	0.66
11B	0.54	7	0.64
13A	0.52	6	0.66
15	0.51	5	0.74
34	0.51	5	0.73
14	0.46	5	0.66
27	0.33	4	0.55
28	0.28	5	0.41
31	0.28	4	0.46
7	0.12	2	0.40

**H*′, Shannon–Wiener diversity index (calculated with log_10_). *S*, richness; *J*′, evenness. Samples are ordered based on decreasing *H*′ values.*

### CLUSTER ANALYSIS OF *THALASSIOSIRA* SPECIES DISTRIBUTIONS

**FIGURE 5:**
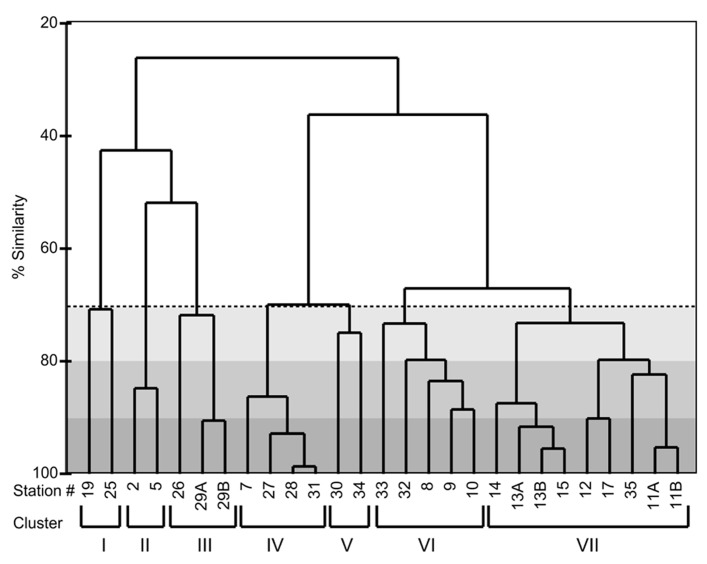
**A cluster dendrogram showing the Bray–Curtis similarity of the ITS2 region fragment analysis from field samples.** The dashed line indicates the ≥70% similarity cut-off. The light gray shaded region indicates ≥80% similarity and darkest gray ≥90% similarity. The roman numerals at the bottom of the dendrogram refer to clusters of stations that are ≥70% similar to each other. Descriptions are as follows: I. OSP; II. Vancouver Coast; III. Haida Gwaii shallow; IV. Haida Gwaii deep; V. Cape St. James/former center of eddy; VI. Between Haida eddy and coast; VII. Haida eddy core and oceanic edge.

A cluster dendrogram of the Bray–Curtis similarity of the relative abundance of ITS2 fragments shows that the ARISA pattern is highly reproducible among replicate samples (**Figure 5**). Replicate filtration was conducted at stations 11, 13, and 29 these samples differed only by ≤10%. In some cases, there were also high similarities in ARISA patterns (≤10% difference) among different stations (e.g., stations 27, 28, and 31; stations 12 and 17; and stations 13 and 15; **Figure 5**). Samples from the OSP collected 4 days apart formed a cluster that was ≥70% similar in *Thalassiosira* species composition (cluster I, **Figure 5**). Cluster II groups at ≥80% similarity and was composed of two coastal stations (2 and 5) that were sampled at the same location 30 h apart (**Figure 5**). Cluster III is comprised of station 26, where southward advecting shelf water was squeezed against the northwest coast of Haida Gwaii, and station 29, which is on the inshore path of this southward advecting shelf water and groups together with ≥70% similarity in *Thalassiosira* composition (**Figure 5**). Stations located adjacent to the shelf break in deeper waters (stations 7, 27, 28, and 31) formed a cluster that was ≥80% similar in *Thalassiosira* species composition (cluster IV, **Figure 5**). The *Thalassiosira* species composition at station 30 off Cape St. James, near the likely origin of Haida eddy surface waters, was ≥70% similar to that found at station 34, which had been the location of the center of the eddy when we first sampled it (cluster V, **Figure 5**). Stations inshore of the eddy but outside the shelf break (stations 9–10 and 32–33) formed another cluster that was ≥70% similar in *Thalassiosira* community composition (cluster VI, **Figure 5**). All of the samples during the first excursion through the Haida eddy, plus the sample from the center of the eddy when we returned 2 weeks later were ≥80% similar in *Thalassiosira* species composition (stations 11, 12, 17, and 35; **Figure 5**). Stations at the leading edge of the Haida eddy (stations 13–15) were ≥80% similar in *Thalassiosira* species composition and grouped with the samples from the eddy core with ≥70% similarity (cluster VII, **Figure 5**).

### ANALYSIS OF CORRELATION BETWEEN ENVIRONMENTAL VARIABLES AND *THALASSIOSIRA* COMMUNITY COMPOSITION

**FIGURE 6:**
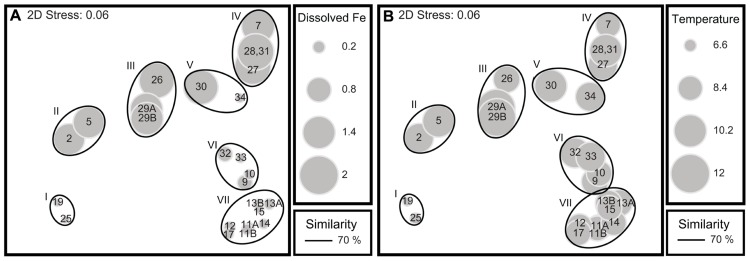
**(A,B)** Multidimensional scaling plots of the Bray–Curtis similarity of the fragment analysis profiles of the ITS2 amplicon composition from field samples. The solid lines encircling various groups of markers correspond to the 70% similarity line on the Bray–Curtis dendrogram and the roman numerals and descriptions labeled on **(A)** are the same as those in **Figures 4** and **5**. Stations 8 and 35 are not included in the MDS analysis, as dissolved Fe was not measured at those stations. **(A)** The size of each individual gray marker corresponds to the surface (10 m) dissolved Fe value measured at that station. **(B)** The size of each individual gray marker corresponds to the sea surface temperature value measured at that station. All corresponding environmental data is listed in **Table 4**.

The Bio–Env correlation with community dissimilarities included sea surface values for temperature, salinity, chlorophyll fluorescence, and dissolved Fe concentrations as well as bottom depth (**Table 4**). Sea surface temperature was highest at the coast, decreasing offshore, and was the lowest at OSP. Salinity was lowest at the coast, increased offshore and was highest at OSP. Dissolved Fe was high at the coastal and shelf break stations, dropped significantly as we moved offshore, and was the lowest at OSP. Fluorescence was more varied, but was generally highest at the coast and lower as we moved offshore. The Bio–Env correlation that was the strongest with the relative abundance data for the fragment analysis occurred with the following combination of environmental data: temperature, fluorescence, and dissolved Fe (ρ_s_ = 0.664, *p* ≤ 0.001). The next strongest correlation (ρ_s_ = 0.656, *p* ≤ 0.001) was a combination of the above variables and salinity, and the third strongest correlation (ρ_s_ = 0.635, *p* ≤ 0.001) was a combination of temperature, dissolved Fe, and salinity. To determine the role that correlation between environmental variables may be contributing to the Bio–Env correlation, we ran a non-parametric (Spearman) correlation of each environmental variable against all the other environmental variables. All environmental variables were significantly positively or negatively correlated with one another and all had their strongest correlation with bottom depth and weakest correlation with fluorescence (**Table 5**). Both temperature and dissolved Fe were strongly negatively correlated with salinity and strongly correlated with each other. Removing bottom depth as a variable from the Bio–Env analysis had no effect as bottom depth did not prove to be an important variable in influencing community composition. Removing either salinity or fluorescence as variables still results in strong Bio–Env correlations (ρ_s_ = 0.635 and 0.664, respectively), thus we are unable to distinguish which of the two correlated variables, fluorescence or salinity, is driving the relationship between the environmental variables and the *Thalassiosira* community composition. Removing either temperature or dissolved Fe as a variable from the Bio–Env analysis, however, does impact the correlation dropping the strongest correlation to ρ_s_ = 0.58 and 0.59, respectively. Thus, it appears that temperature and Fe individually contribute as factors impacting *Thalassiosira* community composition. Taking all of these correlations into consideration, it appears that temperature and dissolved Fe were the quantitatively most significant drivers in the shifts in *Thalassiosira* community composition amongst the factors we measured. Overlaying the dissolved Fe values on an multidimensional scaling (MDS) plot of the similarity of fragment distributions between the different stations illustrates the linkage between dissolved Fe concentrations and Thalassiosiroid community structure; many of the low Fe stations group together and many of the high Fe stations group together (**Figure 6A**). Overlaying the sea surface temperature values on the same MDS plot, also highlights the role that temperature might be playing in driving shifts in *Thalassiosira* assemblages particularly at OSP (**Figure 6B**).

**Table 4:** Surface values of environmental parameters used in BVSTEP analysis.

Station	Sea surface temperature (ITS-90)	Salinity (PSS-78)	Fluorescence (RFU)	Dissolved Fe (nM)	Bottom depth (m)
2	9.45	30.6	4.06	1.30	209
5	10.1	30.1	4.03	1.30	209
7	8.45	31.9	1.17	1.45	2260
9	8.89	32.1	1.56	0.30	2610
10	8.61	32.2	1.49	0.14	2668
11	8.47	32.1	0.27	0.16	2692
12	8.53	32.1	0.45	0.17	2906
13	8.84	32.2	1.00	0.16	3005
14	8.55	32.2	0.41	0.13	3191
15	8.49	32.2	0.18	0.12	3379
17	8.59	32.1	0.13	0.16	2692
19	6.34	32.6	0.65	0.08	4226
25	6.49	32.6	0.50	0.08	4226
26	8.85	31.8	2.28	1.64	352
27	9.42	32.1	1.93	1.73	768
28	9.60	32.1	0.94	1.68	1547
29	11.7	30.7	0.70	1.40	252
30	10.2	31.7	1.02	1.59	2138
31	10.2	32.0	1.56	1.76	907
32	9.59	32.1	1.18	0.30	2926
33	9.45	32.2	1.26	0.12	2608
34	9.37	32.1	0.28	0.11	2691

*Dissolved Fe values from station 7 (as CSJ), station 11 (as EC), station 15 (as O2), and station 19 (as OSP) were previously published in Xiu et al. (2011). The strongest correlation between ARISA Bray–Curtis similarity values and environmental variables was achieved by a combination of temperature, fluorescence, and dissolved Fe (ρ_s_ = 0.66).*

**Table 5:** Spearman’s correlation coefficient (ρ) and associated *p* values showing the highly significant positive and negative correlations observed between the environmental parameters measured at the different stations.

	Temperature		Salinity		Fluorescence		Dissolved Fe	
	ρ	*p*	ρ	*p*	ρ	*p*	ρ	*p*
Salinity	-0.69	0.0001
Fluorescence	0.49	0.01	-0.51	0.009
Dissolved Fe	0.62	0.0009	-0.77	<0.0001	0.54	0.0046
Bottom depth	-0.73	<0.0001	0.92	<0.0001	-0.62	0.0009	-0.76	<0.0001

## DISCUSSION

Our results clearly associate shifts in the community composition of *Thalassiosira* species across a Haida eddy. Our results also identified temperature and dissolved Fe as factors driving shifts in *Thalassiosira* community composition in the northeastern Pacific Ocean. Recent work indicates that Haida eddies may be contributing as much Fe to the Gulf of Alaska as atmospheric dust deposition does on an annual basis (Xiu et al., 2011). Haida eddies have previously been associated with both increased chlorophyll concentrations and changes in phytoplankton community composition (Crawford et al., 2005; Peterson et al., 2011a,b), potentially because they mix coastal waters having high Fe concentrations with HNLC waters high in macronutrients (Johnson et al., 2005; Xiu et al., 2011). In particular, biological productivity and diversity along the leading edge of eddies appears to be stimulated by mixing water masses combining high nutrients outside the eddy with elevated Fe within the eddy (Peterson et al., 2011b). Another hotspot of phytoplankton diversity and primary productivity in the northeastern Pacific Ocean is the transition zone between coastal and offshore waters, where high Fe coastal water mixes with nutrient rich open ocean water typical of this HNLC region (Ribalet et al., 2010), supporting the idea that Fe from coastal waters is an important driver of biological activity in the region.

### VERIFICATION OF PRIMER SPECIFICITY TO *THALASSIOSIRA* SPP.

Our PCR primers proved successful at amplifying the ITS2 region from mixed assemblages of *Thalassiosira* in environmental samples. While the primer pair was designed to target *Thalassiosira* spp., the ITS2 rDNA *Skeletonema* spp. and *Minidiscus* spp. sequences in GenBank^®^ differ from *Thalassiosira* spp. by only one to two bases in the primer region. Given that *Skeletonema* spp. and *Minidiscus* spp. often group within *Thalassiosira* spp. in phylogenetic trees based on rDNA cistron components (Kaczmarska et al., 2005; Alverson et al., 2007), it is not surprising that we obtained sequences from both of these closely related lineages, especially in regions where *Skeletonema* and *Minidiscus* were likely a major proportion of the biomass, as at the coastal stations (Aizawa et al., 2005; Kaczmarska et al., 2005; Buck et al., 2008). Examination of the *Chaetoceros* spp. ITS2 sequences in GenBank^®^ shows multiple mismatches to our primers for the majority of the *Chaetoceros* species. *Chaetoceros atlanticus* has the most similar sequence to our primers with one mismatch to the forward primer and five mismatches to the reverse primer (all at the 5′ end of the primer). Given the multiple mismatches between our primers and the *Chaetoceros* sequences in GenBank^®^, any significant recovery of *Chaetoceros* sequences suggests that *Chaetoceros* cells greatly outnumbered *Thalassiosira* cells in a given sample, as was likely the case at OSP. This conclusion is supported by analysis of diatom community composition with shipboard light microscopy of OSP samples. Few *Thalassiosira* were identified, but multiple *Chaetoceros* spp. were present at OSP (L. Whitney, personal observation). In designing the primer set, efforts were made to ensure the primers would amplify all *Thalassiosira* spp. present in a sample, acknowledging that some non-*Thalassiosira* spp. might be amplified. Given that the two non-*Thalassiosira* spp. most likely to be amplified by the primers group phylogenetically with the *Thalassiosira* genus, the retrieval of those sequences should not be deemed a failure of the primers, but rather an indication that the primers provide a useful tool for ascertaining relative changes in *Thalassiosira* species and close relatives in mixed assemblages.

### ARISA AS A TOOL FOR IDENTIFYING SPECIFIC *THALASSIOSIRA* SPECIES

Microscopic examination of samples revealed the presence of small unidentified *Thalassiosira* spp. throughout the cruise with the exception of samples from OSP and that small *Thalassiosira* spp. were especially prevalent on the transect off of Haida Gwaii (T. Rynearson, unpublished). An advantage of our ARISA method is its ability to identify species that are otherwise difficult to classify by standard approaches.

Both the ARISA profiles and the sequence library demonstrate that *T. oceanica* is a significant component of the *Thalassiosira* community in the northeastern Pacific Ocean at the time we sampled, although it was not found at OSP. ARISA analysis indicated that *T. oceanica* dominated *Thalassiosira* community composition in the open ocean stations associated with the leading edge of the Haida eddy (stations 13–15). Although *T. oceanica* often is considered to be a warm water species (Tomas, 1997; Kaczmarska et al., 2005), a number of studies report finding *T. oceanica* in waters as cold as 12°C (Harris et al., 1995; Aizawa et al., 2005; Garcia and Odebrecht, 2009) and the original description of cultivated *T. oceanica* maintained growth at 12°C (Hasle, 1983). The cruise occurred very shortly after the spring bloom at OSP and water temperatures were 2°C colder at OSP than at the other stations, which may have contributed to the absence of *T. oceanica* there. The presence of *T. oceanica* at the shallow coastal stations may seem out of place for this originally described oceanic species, but *T. oceanica* has previously been found in coastal environments (Harris et al., 1995; Aizawa et al., 2005; Garcia and Odebrecht, 2009). The small size of *T. oceanica* makes it challenging to identify by light microscopy, especially when larger diatoms are a more prominent component of biomass, thus it might be poorly enumerated in coastal stations.

*Minidiscus* spp., another genus of the Thalassiosiraceae that is often missed in microscopic analyses because of its small size (Kaczmarska et al., 2009), was also shown to be a significant component of the *Thalassiosira* community in both coastal and oceanic samples, except those at OSP. Previous studies using electron microscopy have shown *Minidiscus* spp. to be very abundant in this region, particularly near the coast (Aizawa et al., 2005). The requirement for electron microscopy for identifying *Minidiscus* spp. means that knowledge about their distribution throughout the world’s oceans is limited (Tomas, 1997). *Minidiscus* spp. seem to be closely associated with coastal and upwelling environments (Aizawa et al., 2005; Buck et al., 2008), findings our data support.

The sequence analysis revealed a novel *Thalassiosira* sp. with an ITS2 length of 367 bases that is also a diagnostic ARISA fragment length (group “d”: **Figure 2**). Fragments of this unique length were detected at low levels in coastal samples, and were a significant portion of the fragments detected in the center of the Haida eddy (stations 11, 17, and 35), and similar to *T. oceanica* and *Minidiscus* spp., were not prevalent at OSP. It is possible that this sequence is associated with either *T. eccentrica*, *T. nordenskioeldii*, or *T. pacifica*, species previously shown to be important in the region (Aizawa et al., 2005) but for which ITS2 sequences in GenBank^®^ are lacking. Light microscopy on samples from the cruise showed that *T. eccentrica* and *T. nordenskioeldii* were common along the cruise track (T. Rynearson, unpublished). Alternatively, this fragment could be associated with an unknown or uncultured *Thalassiosira* spp. As the number of ITS2 sequences in public databases increase, we may be able to identify this key player in the future.

The “model” *Thalassiosira* species used regularly in laboratory studies include *T. pseudonana* and *T. weissflogii* but neither were detected in these analyses, and they are not reported to be common in temperate offshore phytoplankton assemblages (Haigh et al., 1992; Aizawa et al., 2005; Hoppenrath et al., 2007). These findings highlight that the predominant *Thalassiosira* species in the northeastern Pacific Ocean are not organisms that are typically targeted in functional genomic laboratory studies, with the exception of *T. oceanica*, which has recently been included in genomic studies (Lommer et al., 2010, 2012). One of the benefits of the ARISA assay is that it can help to identify *Thalassiosira* species that are important players in different oceanic regions, which, in turn, can help motivate future laboratory physiology and functional genomic studies to better constrain their physiology and biogeochemical roles in the environment.

### *THALASSIOSIRA* SPECIES DIVERSITY IN THE NORTHEASTERN PACIFIC OCEAN

While our sequencing efforts were not done with the goal of evaluating the diversity of the *Thalassiosira* in the region, we are encouraged by the general agreement of sequence data with ARISA data. Not surprisingly, since sequencing per sample was not saturating, ARISA identified more species than did sequence analysis. In most cases where this occurred, multiple small ARISA peaks were observed in samples where only one sequence type, usually associated with the dominant peak, was detected in the sequence library from that station. Our goal in generating the sequence library was to be able to identify fragments that are diagnostic in length, not to sequence to rarefaction on each sample. These results serve to highlight an advantage of the ARISA method, the ability to detect even low abundance sequence types in a given seawater sample.

Both enumeration of ARISA peaks and statistical measures support our conclusions that the shallow coastal stations were more diverse than the other stations and the samples from the shelf break by Haida Gwaii were the least diverse. The increased diversity at the coastal stations is consistent with higher dissolved Fe concentrations in these waters, likely also enriched with macronutrients. The low diversity at the samples from the shelf break is interesting, as these stations also had high dissolved Fe and chlorophyll *a* fluorescence values. Previous work in the region has found that the shelf break is associated both with high productivity and high diversity (Ribalet et al., 2010). The shelf break stations in our study have high fluorescence, which is consistent with it being a highly productive region, while our ARISA results indicate low *Thalassiosira* diversity. The low diversity in these samples could indicate a mono-specific *Thalassiosira* bloom, which would be consistent with both our ARISA data and fluorescence measurements. Alternatively, it could be that phytoplankton species that are not part of the *Thalassiosira* genus, which cannot be detected with this method, are abundant at the shelf break. This method, while useful in comparing the relative proportions of different *Thalassiosira* spp. between samples, does not provide information on the absolute abundance of individual species and provides no insight into the overall diversity of the phytoplankton community.

### CLUSTER ANALYSIS OF *THALASSIOSIRA* SPECIES DISTRIBUTION

Interestingly, cluster analysis of the *Thalassiosira* species composition patterns in each sample identified discrete clusters corresponding to geographical regions that are likely to have distinct oceanographic properties. One such cluster included the stations along the coast of Haida Gwaii, which were potentially influenced by recent shelf waters advecting along the outer northwest margin of the island. Another cluster grouped stations from the shallow shelf off the northern tip of Vancouver Island. Two separate clusters grouped stations in the region just offshore of the shelf break and the two samples from OSP taken four days apart.

Many of the Haida eddy stations were grouped in clusters; consistent with recent data showing that there are significant shifts in phytoplankton community composition associated with the edges of Haida eddies (Crawford et al., 2005; Peterson et al., 2011b). These data show distinct separation in Thalassiosiroid community composition at the leading and trailing edges of the westward advecting eddy, with the trailing edge being more similar to nearshore waters, while the leading edge community was more aligned with that of the eddy core. The juvenile Haida eddy in 2007 had only separated from the shelf break near Cape St. James 3 months earlier (Xiu et al., 2011), and so it is not surprising that the Thalassiosiroid community in the eddy core surface waters showed some similarity with shelf waters likely along the eddy’s path. Batten and Crawford (2005) found shelf species of diatoms (*Thalassiosira* spp., *Chaetoceros* spp., *Coscinodiscus* spp., and *Cylindrotheca closterium*) and calanoid copepods within or closely associated with Haida 2000 and 2001, consistent with our characterization of Thalassiosiroid distributions. That Thalassiosiroid diversity in stations within the eddy is more similar to that in oceanic waters than coastal waters could be the result of the complex patchiness of surface waters generated by dynamic advective upwelling and downwelling processes in the eddy (Xiu et al., 2011).

Another interesting feature of the Haida stations clustered by their similar Thalassiosiroid communities is that station 35, which was the new center of the eddy core on the return trip 2 weeks after the initial sampling, groups with the other eddy core stations. Meanwhile, station 34, which was at the same geographic location as the original eddy core (stations 11 and 17), does not cluster with the eddy-associated stations. These findings demonstrate how ARISA analysis provides unique insights to tease apart differences among diatom communities or in this case, the differences in community trajectories as influenced by oceanographic conditions.

### Fe AND TEMPERATURE AS DRIVERS OF SHIFTS IN *THALASSIOSIRA* COMMUNITY COMPOSITION

Analyzing Thalassiosiroid distributions in the context of other environmental variables revealed correlations that may represent drivers controlling *Thalassiosira* spp. distributions in the northeastern Pacific Ocean. The environmental variables that were considered were temperature, salinity, chlorophyll *a* fluorescence, bottom depth, and dissolved Fe concentrations. Chlorophyll *a* fluorescence was included as an environmental variable as it is a bulk measurement that represents the whole phytoplankton community, while our assay provides information on a subset of the chlorophyll-containing phytoplankton community, the *Thalassiosira* diatoms. Bottom depth was included as numerical way to distinguish between coastal, shelf, and open ocean sites. Removing it from our statistical analyses does not change the results. Unfortunately, since macro nutrient concentrations were not measured for every station and we did not measure the concentrations of other potentially important micronutrients for diatoms such as zinc and B vitamins, we could not consider them in our statistical analysis. The three strongest correlations (ρ_s_ ≥ 0.635) between environmental variables and Thalassiosiroid community composition all included dissolved Fe and temperature as variables contributing to describe the phytoplankton community constraints at each station. Previous work in a region just north of where our samples were taken found that macronutrient concentrations (NO_3_^-^ and PO_4_^-^) and vitamin B_12_ concentrations inversely correlate with dissolved Fe (at *R* = -0.75, -0.84, and -0.49, respectively; Koch et al., 2011), thus implying that if we had measurements of these variables, they might also correlate with the changes in Thalassiosiroid community composition. Because of the correlation between dissolved Fe and temperature, it is hard to distinguish the contributions that each variable may have imparted on *Thalassiosira* community composition. It may be that one or both of these environmental factors impart a driving force on *Thalassiosira* community composition in concert or each does fractionally. The marked correlation with dissolved Fe is likely to have physiological significance given the wide range of Fe requirements among the few *Thalassiosira* spp. studied (Sunda and Huntsman, 1995). Dissolved Fe is known to be a limiting nutrient for phytoplankton productivity in the region (Boyd et al., 2004; Johnson et al., 2005), and it is suggested to be a primary reason why Haida eddies are so productive (Crawford et al., 2005; Peterson et al., 2011b; Xiu et al., 2011). While our data supports previous work showing that Fe is an important driver of biological processes in the region, temperature was also correlated with changes in *Thalassiosira* community composition. This is fitting with our hypothesis that the colder waters sampled at OSP were a barrier for *T. oceanica* growth.

In conclusion, we have developed a method for rapidly identifying *Thalassiosira* spp. in environmental samples. The method is particularly useful at identifying some of the smaller *Thalassiosira* spp. that are difficult to distinguish using light microscopy, though it lacks in the ability to definitively identify some of the larger species. This method was used to determine the community composition of *Thalassiosira* species in samples from the northeastern Pacific Ocean, where microscopic inspection of samples had revealed small centric diatoms were a significant component of the planktonic biomass. The method was also able to link some of the shifts in species distribution to the presence of a 3-month-old Haida eddy and the Fe that it brought to HNLC waters. The potential benefits of this method include the ability to monitor changes in species composition in response to ecological changes, as well as the ability to screen samples for individual species for which we have molecular tools to monitor the genetic response to changing environmental variables. Using species-specific molecular techniques could enable us to move beyond simply correlating shifting community composition with Fe values, as we did here, to assessing Fe limitation of individual species *in situ*.

## Conflict of Interest Statement

The authors declare that the research was conducted in the absence of any commercial or financial relationships that could be construed as a potential conflict of interest.
